# Institutional straddling: Negotiating micro-governance in Hanoi’s new urban areas

**DOI:** 10.1177/23996544211055471

**Published:** 2022-01-27

**Authors:** Danielle Labbé, Gabriel Fauveaud

**Affiliations:** School of Urban Planning and Landscape Architecture, 5622 Université de Montréal, Montreal, QC, Canada; Department of Geography, Center for Asian Studies, 141639 Université de Montréal, Montreal, QC, Canada

**Keywords:** Neighborhood governance, privatization, real estate, homeowner association, Hanoi, Vietnam

## Abstract

The rise and spread of commercially built residential projects in East and Southeast Asia has attracted growing scholarly attention since the 1990s. This scholarship has notably explored how the private production of new residential developments has altered urban governance across the region. However, few studies have analyzed how private corporate actors’ new roles in city-making processes shape governance logics at the neighborhood scale. This paper begins to fill the gap by exploring the governance dynamic of three new urban areas (NUAs) built on the edge of Hanoi in the late 1990s and early 2000s. Our analysis builds on a conceptualization of local state-linked organizations in East and Southeast Asia as “straddlers” Relying on interviews with local community leaders and homeowners, we find that the privatization of urban space production has not sidelined residents and government from micro-local politics in Hanoi. Instead, conflicts between homeowners and developers over land uses, property rights, and management practices and responsibilities have led to a repositioning of the old socialist neighborhood administrative apparatus to accommodate the governance of NUAs. The self-protection response of NUA homeowners during these conflicts played a key role in this process. By deploying what we call institutional straddling, these people forged a new mode of neighborhood governance that weaves together the neighborhood administration apparatus inherited from socialism and new residential property self-management bodies.

## Introduction

Beginning in the early 2010s, the domestic press in Vietnam paid increasing attention to a new kind of neighborhood conflict surfacing across the country’s largest cities (e.g., Ha Noi Moi, 2012). Local conflicts were nothing new in urban Vietnam (Koh, 2006; Papin, 1997); yet in these specific instances, they featured novel socio-spatial configurations: large-scale residential developments locally known as new urban areas^1^ (hereafter NUAs), which had emerged around Vietnamese cities in the late 1990s. In a departure from the past, strife in these master-planned communities did not pit local residents against each other or against local governments. Instead, they were characterized by middle-class households opposing commercial property developers and their property management companies.

This paper takes these new neighborhood conflicts in Vietnamese cities as a site for understanding how governance operates in the new, private developments characteristic of East and Southeast Asia’s contemporary urban expansion. Critical scholars have argued that commercially built residential projects, such as Vietnam’s NUAs, have fostered the diffusion of neoliberal and market-oriented urban development politics throughout the region (e.g., Shatkin, 2017). The involvement of private businesses in the production of new urban neighborhoods has also been associated with a retreat of the state from its traditional urban governing functions in favor of more private forms of urban administration (e.g., Dieleman, 2011; Fauveaud, 2015; Ortega, 2012; Pow, 2009).

These arguments stem mainly from studies of higher-end gated communities and guarded residential developments concerned with their exclusionary and segregative impacts (e.g., Harms, 2016; Roitman and Recio, 2020; Tedong et al., 2015; Zhang, 2010). Much less attention has been paid to the vast number of mid-range and often non-gated residential projects developed in and around East and Southeast Asian cities such as Hanoi. What kinds of relations between new homeowners, property developers and managers, and local governments have been forged in these neighborhoods? And what forms of governance ensue from these relations?

This paper begins to answer these questions through an analysis of the micro-local governance dynamic in three NUAs located at the near periphery of Hanoi—Linh Dam, Trung Hoa-Nhan Chinh, and Van Quan. These cases suggest that commercially built residential projects in the Vietnamese capital did not lead to sidelining local residents and government from micro-local politics. Instead, we observed in these three NUAs a repositioning of pre-existing actors in the local governance structure. This repositioning is closely connected to a mode of political action fusing old and new neighborhood administrative institutions. Homeowners in these NUAs have initiated and deployed this strategy to collectively defend their property interests in the face of problematic urban administration practices by developers and their property managers.

All three NUA projects were built by state-owned property developers^2^ in the late 1990s and early 2000s and had been inhabited for at least a decade by the time of our investigation. The vast majority of households in these projects are Vietnamese and belong to the emerging professional and middle classes. Between 2012 and 2017, we conducted approximately 60 semi-structured interviews with residents, focusing on owners of apartments in high-rise buildings. As will be discussed below, this NUA housing type has been the locus of significant governance-related tensions. Our analysis centers on a subset of 20 interviews with apartment owners who have been actively involved in disputes with NUA developers and property managers. These were complemented with a policy review and a dozen interviews with developers, state planners, and architects involved in the formulation and implementation of the NUA model in Vietnam.

The body of this paper is organized as follows. Section 1 reviews the literature on commercially built residential projects in East and Southeast Asia with a focus on the ways in which it has addressed urban governance issues. It also introduces the concepts of institutional straddling mobilized for our analysis. Section 2 describes the context in which NUA projects developed in Vietnam and discusses the main policies governing their management and governance. Section 3 analyses the conflicts that emerged between homeowners and developers in the three sites studied and how owners defended their interests by weaving together old and new neighborhood administration bodies.

## Section 1: Micro-governance of commercially built residential projects in East and Southeast Asia

While the private sector’s involvement in urban space production has a long history in East and Southeast Asia (Hogan et al., 2012; Philips and Yeh, 1987), the 1990s marked a turning point. As in the rest of the world, this period saw accelerated private sector-led urban space production and, at the periphery of cities, the diffusion of new, more or less enclosed urban configurations such as gated communities, new towns, and satellite cities (see Keeton, 2011; Shatkin, 2017).

This enclave urbanism movement spurred significant governance changes. A number of macro-scale transformations have been reported in several areas of East and Southeast Asia. The transfer of planning responsibilities from the public to the private sector, for instance, seems to have fragmented territorial governance and eroded regional planning in places as diverse as Cambodia, Vietnam, and Indonesia (see Fauveaud, 2017a; Firman, 2004; Labbé, 2019; Paling, 2012). Recent, localized studies, however, point toward more nuanced effects of privatized urban space production on governance. These local dynamics are more situated insofar as they take form through interactions with specific political regimes, institutional legacies, urban development policy frameworks, regulatory practices, and political struggles (e.g., Lu et al., 2020; Tedong et al., 2015).

These ideas echo the argument of Hogan et al. (2012) that private development in Asia underpins a range of urban citizenship transformations, each founded on localized interactions between the state, private stakeholders, and urban residents. It follows that understanding the forms of governance emerging in new, privately developed residential areas means attending to both the complex imbrications of private and public institutions and the range of actors with stakes in these neighborhoods (Fauveaud, 2017b). Moreover, while taking seriously the argument that states play a key role in the privatization of urban space production in the region (Shatkin, 2017), one may assume that socio-political life in privately produced spaces cannot totally break with pre-existing governance structures. This raises two important questions: How do pre-existing and new logics of local governance interact with each other in private, mid-range residential projects? And what roles do local residents play in these interactions?

Despite the rapid rise of NUAs in Vietnam in the last few decades, these questions have received scant scholarly attention (but see Huong and Hang, 2018; Huong and Sajor, 2010). The literature however indicates that local residents outside Vietnam can—and often do—play an active role in forming and transforming the local governance of new residential developments. This role appears to be especially salient in socialist and post-socialist contexts where states have transferred significant urban management responsibilities to local populations since the 1980s (e.g., Bray, 2006; Lu et al., 2020; Stanilov, 2007).

In new housing developments, this increased involvement of residents often occurs through homeowner associations (hereafter HOAs), broadly defined as “mandatory membership organizations that make and enforce rules, collect assessments from all owners, manage the common property, and therefore, function as private governments” (McKenzie, 2006, cited in Huong and Sajor (2010): 20). In Asia, research on HOAs’ role in commercially built residential projects has centered on China. This literature links changes in China’s residential local governance system to the housing reforms of the 1980s and 1990s. Through these reforms, the Chinese state gave up its role as primary urban housing producer and manager, privatized state-owned urban housing stocks, invited corporate actors to get involved in housing production, and gave local residents’ groups (called Residents’ Committees) more responsibility and autonomy (see Breitung, 2012; Lu et al., 2020; Yip, 2012). This opened the way for thousands of commercially built residential projects across China and the organization of their homeowners into new, neighborhood-scale residents’ associations (Read, 2003; Tomba, 2014).

As will be discussed in the next section, starting in the 1990s Vietnam adopted housing reforms in many ways similar to those of China, including policies encouraging a local version of HOAs in NUAs. Although there are significant differences between the two contexts, the literature on Chinese HOAs offers useful insights for the study of their Vietnamese counterparts. This literature on the socio-political dynamic of new housing developments notably calls attention to the influential roles played by social stratification (especially middle- and upper-middle-class formation); the rise of new consumer rights discourses and demands; and the emergence of powerful property developers with strong political connections (e.g., Read, 2003; Tomba, 2014; Wang, 2010).

The literature on China further highlights regular disputes in new commercial developments, pitting middle-class residents against private developers over communal land use and property management (e.g., Read, 2003; Tomba, 2014; Zhang 2010). Throughout China, such conflicts have triggered forms of “self-protection activism” among homeowners who seek to defend their shared place-based interests and property rights against developers who “act as a new form of authority beyond the scope of market activities” (Zhang, 2010: 11). The literature shows that homeowner associations play a key role in such self-protective reaction by becoming a vehicle for collective action. Studies further suggest that the ways in which homeowners use HOAs to defend their interests in opposition to developers (and their property managers) shape the governance dynamics in China’s new residential enclaves in meaningful ways (see Read, 2003; Tomba, 2014; Wang, 2014; Wang et al., 2012). This points to the importance of attending to HOAs’ policy frameworks, the interests driving their actions, and how they go about pressing their claims in interaction with other actors and institutions.

With these ideas in mind, we sought a conceptual frame for analyzing forms of local governance that have emerged in Hanoi’s NUAs, bearing in mind the specific history, institutional legacies, and political culture of the Vietnamese capital. Focusing on the ways in which NUAs are managed, we noticed that a hybrid form of governance seems to have emerged in these neighborhoods. It combines local socio-political organizations inherited from socialism with new self-management bodies. Homeowner organizations, we further noticed, not only play a key role in the resulting dynamic but also rely on an intriguing mode of political action that blurs conventional boundaries between the different local administration bodies active in NUAs. More specifically, these actors utilize what we call a form of “institutional straddling” to defend their interests vis-à-vis developers and property managers who have built and continue to administer their NUAs.

The notion of institutional straddling builds and expands on political scientists Read and Pekkanen’s (2009) concept of “straddler organizations,” which “have extensive presence at the grassroots and engage widespread participation, yet are institutionally linked to the state rather than independent of it” (Read, 2009: 1). While straddler organizations appear in various parts of the world, according to Read they are especially prevalent in East and Southeast Asia, including Vietnam (Read, 2009: 10–11). Examples of these local bodies in the region range from statist mass organizations set up by communist states, to state-mandated residents’ committees, all the way to self-constituted volunteer organization and NGOs.

Two interrelated characteristics of straddler organizations, as defined by Read and Pekkanen (2009), are especially useful for grasping the dynamic at play in Hanoi’s NUAs. First, straddler organizations tend to be put to use by both state *and* local communities. More repressive states, such as Vietnam’s communist party-state, tend to use local organizations as mechanisms of population control and as “transmission belts,” aimed at extending the reach of their authority to individual households (Read and Pekkanen, 2009: 4). Yet, Read argues, these same organizations often serve local communities as well, by providing them with services, by acting as communication channels with authorities, and, at least in some cases, by disciplining and holding these same authorities accountable (Read and Pekkanen, 2009: 5). This double role of local, state-mandated administrative bodies is well documented in the context of Vietnam (see, for instance, Kerkvliet and Marr, 2004).

According to Read (2009), one way straddler organizations acquire this double role is by becoming nexuses of neighborhood associational life. In many parts of East and Southeast Asia, he remarks, “[c]ommunity networks build themselves around the nucleus that is established by government structuring” (Read, 2009: 13) This fusing process can—and often does—allow local communities to use local bodies set up by the state for purposes markedly different from those originally intended. “What seem to be tame organizations embedded within powerful states,” Read writes, “can in fact slip their moorings and undertake feisty activities that their sponsors never intended” (Read, 2009: 6). We contend that a similar phenomenon is at play in Hanoi’s NUAs. Homeowner groups are diverting state-linked micro-local administration bodies from their original functions, using them instead to defend their place-based and private property interests.

A second related key characteristic of straddlers is that they can—and often do—“meld conceptually distinct functions, so that the boundaries between them are blurred” (Read, 2009: 13). Read and Pekkanen (2009) are primarily concerned with the ways in which local organizations blur the state-society divide in East and Southeast Asia. In what follows, however, we argue that the mode of political action adopted by groups of NUA homeowners in Hanoi blurs more than the state and non-state boundary. It also purposefully intertwines neighborhood administration organizations established by the socialist state with new, self-management bodies reflecting private property.

## Section 2: NUA micro-governance: Between corporate and late-socialist systems

### The NUA model of urban development and the shift in housing production

The NUA model of urban development emerged in the 1990s in of the wake of Vietnam’s *doi moi* reforms.^3^ Commenting on the profound institutional changes that characterized this period, a planner at the Ministry of Construction recalled: “The reforms forced us to move on, to find alternative tools to manage urban space. We could not conduct housing and investment activities as we had done under centralized planning (*ke hoach tap trung*). We had to find another way.” (interview 21/12/2016).

In the words of a former Chief Architect of Hanoi, part of “another way” formulated in the early 1990s was “to develop the housing market in the form of big projects” (interview, 11/12/2016). During that period, state planners started to envisage the development of large, self-contained master-planned communities at the periphery of existing cities. This model, later referred to as new urban areas (NUAs), sought to channel non-state capital, including foreign investment, into producing the residential spaces much needed by rapidly growing urban populations. The Vietnamese state further conceived of NUAs as a means to realize an urban (re)territorialization project centered on ideals of orderly (*trat tu*), modern (*hien dai*), and civilized (*van minh*) cities fit for a developed nation in the making (see Harms, 2016; Tran and Yip, 2008, 2019).

NUAs have since become an important driver of Vietnamese cities’ expansion. According to a survey conducted by one of the authors, at the end of 2015, the province of Hanoi alone had approved 252 such developments. These projects vary considerably in size (from 10 to 2000 hectares) and planned population (from a few hundred to over 200,000 residents) (Labbé and Musil, 2017). Commercial housing geared toward the growing professional and middle classes dominates projects of less than ten hectares. Larger NUAs may also include private amenities (e.g., gyms, medical clinics, kindergartens), commercial and office spaces, open public spaces, and, occasionally, social or resettlement housing. The majority of projects, while not gated, tend to be partially segregated from their surroundings due to poor road connections, infrastructural barriers, exclusive amenities, etc.

In Hanoi, as in other Vietnamese cities, NUA construction has drastically altered metropolitan formation processes. This goes beyond the dramatic visual impact of large thoroughfares and residential high-rises emerging out of paddy fields. At a deeper level, the adoption of this model of urban development has signaled profound transformations of the urban space production approach upheld by the state since independence. Major policy changes that made the implementation of the NUA model possible included:

- The gradual liberalization of property rights over residential land and housing, followed by sanctioned real estate markets;
- A broadening of the state’s power to confiscate use rights over peri-urban agricultural lands and to transfer them into private hands for urban redevelopment, leading to the monetization and commodification of this public resource;
- A retreat of the state from residential space planning, production, and allocation in favor of privatized planning and market-based provision (see Labbé, 2016; Labbé and Musil, 2014; Tran and Yip, 2008, 2019)

These policy changes may appear to be a radical supplanting of the socialist city-building approach by market capitalist mechanisms. But the NUA model neither fully rejects socialist ideals, principles, and institutions nor fully embraces their market capitalist counterparts. Instead, it is the result of a “hybrid system brought about by a complex interaction among the emerging market imperatives amidst the changing socialist legacy” (Tran and Yip, 2019: 115).

State policies, for instance, require that NUAs be functionally self-sufficient and formally uniform (e.g., Hanoi People’s Committee, 1995; Ministry of Construction, 2008), two socialist planning principles inherited from the pre-reform era. Another instance of hybridity is the predominance, in NUA developments, of state-connected actors and entities akin to what Gainsborough (2010: 34) has called “state business interests.” This situation is closely linked to the structuring of Vietnam’s property development sector in the early *doi moi* years. From the start, governments entrusted the implementation of NUAs to state-owned enterprises (interview, 27/12/2016; see also Labbé and Musil, 2014). State-connected developers continued to be prioritized in the following decades and were nurtured by direct links to state agencies and territorial administrations. A survey conducted by one of the authors revealed that, by 2015, half of the NUA projects approved on Hanoi’s territory were being developed by a former state-owned enterprise or by a private business set up by state agencies connected to the army, the police, or to provincial- or district-level local governments. As indicated by a recent study (Hoang, 2018) and by our own interviews, to this day social relationships of patronage with state officials (sustained by bribes and other illegal practices) continue to facilitate politically connected domestic developers’ access to land, credit, investment licenses, and construction permits. We will see below that the blurry business-state frontier characteristic of urban property development in Vietnam is an important source of tensions between homeowners and developers.

### A hybrid micro-governance framework

Another important instance of NUAs’ market-socialist hybridity, which has received limited scholarly attention, concerns governance. Like the rest of Vietnam, the territories and populations of mid-range NUAs such as those on which this project focuses, fall under the socialist political-administrative apparatus inherited from the pre-reform era.^4^ While there have been some transformations since *doi moi* (see Kerkvliet, 2004), this system remains defined by a highly vertical hierarchy of governing institutions reaching all the way down to local communities and individual households (for further discussion see Koh, 2006; Leaf, 1999).

The establishment and maintenance of this system within NUAs is the responsibility of the executive body of ward governments called People’s Committees.^5^ Early on in the lifecycle of new residential developments sited on their territory, the Committees are required to provide them with the socialist neighborhood-scale administrative apparatus. This involves dividing the territory and population of NUAs into residents’ groups (*to dan pho*), establishing local party cells (*chi bo*), and setting up branches of the Fatherland Front’s mass organizations.^6^ In the three NUAs studied, this network of state-mandated organizations was set up between one and three years after the first residents had moved in.

These grassroots bodies display key characteristics of the straddler organizations conceptualized by Read and Pekkanen (2009). Their official mandate is to disseminate and implement the party-state’s policies and campaigns and ensure its social and political control over local households. This is also how leaders of these bodies in the NUAs that we studied understand their roles. Asked about the purpose of the local body he leads in Linh Dam, the head of a residents’ group replied, “It’s the same as everywhere else: we are here to deploy [*trien khai*] policies coming from higher echelons to the local population, to keep residents united [*doan ket*] behind the party, and to ensure security and order [*an ninh trat tu*]” (interview, 20/7/2013d).

In practice, however—and like other straddlers in East and Southeast Asia—Vietnam’s local state-linked organizations regularly take on other roles (see for instance Kerkvliet, 2004). We observed the same phenomenon in the three NUAs studied. There, interviewees reported that residents’ groups and branches of mass organizations play active roles in spheres as diverse as resolving neighborhood-level conflicts, promoting mutual aid, and organizing socialization and recreational activities ranging from children’s festivities, to aerobic and dance classes, to chess and table tennis clubs for the elderly.

Several interviewees mentioned that these bodies play a limited role in the daily lives of many NUA residents, especially compared to their inner-city and rural counterparts. Leaders of these organizations explained this situation by pointing to the predominance of young families in mid-range residential developments. “These young people are out from morning to evening,” one leader remarked. “When they come back home, they have to take care of their children, to cook dinner so they rarely join common activities and, in these circumstances, it is difficult for us to encourage them to do so” (interview, 20/7/2013c). A young professional living in one of Van Quan’s high-rises with his wife and two young children mentioned a similar generational gap. While older homeowners, retirees in particular, are actively involved in his NUA’s local political life, he noted that most people in his age group “don’t know who is the head of their residents’ group or the secretary of their local party cell. They are too busy working” (interview, 16/6/2017).

In addition to the socialist neighborhood administration apparatus, and in contrast to existing urban neighborhoods, the policy framework governing NUAs means that they are dotted with non-state, urban management bodies. The first of these, called management units (*don vi quan ly*) and management boards (*ban quan ly*), must be set up by developers when residents start to move into their projects. Management units operate at the neighborhood scale while boards are typically in charge of smaller areas—for instance, a cluster of two or three residential high-rises. These bodies ensure maintenance of neighborhood infrastructure and amenities (parks, on-street parking, etc.) as well as common spaces and equipment in high-rise buildings (corridors, elevators, etc.). They also provide residents with services such as garbage collection and security. As is the case for common-interest developments found in other parts of the world, policies allow urban management units and boards to charge NUA homeowners a monthly fee for these services (Decisions 08/2008/QD-BXD and 02/2016/TT-BXD, Law on Housing, 2014).

According to legislation governing NUAs, this arrangement should be temporary. Once development construction is complete, the law requires a developer to transfer its public components (streets, parks, utilities, and amenities) to the ward government, which should thereafter manage them as part of its regular responsibilities and services (policing, garbage collection, public space maintenance, etc.) (Decisions 08/2008/QD-BXD). Key informants from the Ministry of Construction and from Hanoi’s municipal government, however, indicated that very few such transfers have taken place on the territory of the Vietnamese capital (interviews, 21/12/2016 and 12/6/2017). While we did not explore this issue specifically, we note that it affords developers a prolonged stranglehold over NUA governance.

Policies also call for changes in the administrative system of high-rise residential towers past their early occupation period. These NUA buildings introduced into urban Vietnam a new form of residential tenure founded on co-ownership, calling for new modes of administration and management. Early policies adopted to this end (Law on Housing 2005, Decision 08/2008/QD-BXD) stipulated that once half of the housing units in residential high-rises were delivered, residents could elect a homeowner association (HOA) called a governance board (*ban quan tri*). Between 2008 and 2016, the legislative framework allowed these HOAs to take over any of developers’ roles and responsibilities for NUA building management, including elaborating internal operation rules, hiring maintenance companies, collecting monthly service fees, and coordinating with local governments (art 12.2 Decision 08/2008/QD-BXD).

In theory, then, HOAs could entirely exclude developers from residential high-rise management. In reality, groups of apartment owners who sought to form HOAs in the three NUAs we studied faced significant resistance from developers and, to a lesser extent, from local governments. Involved residents explained that property developers see HOAs as a threat, notably because they tend to militate for more public and transparent forms of management and, in some cases, seek to appropriate revenues their buildings generate from such things as underground parking and commercial space rental. These same interviewees reported that local governments at the ward, district, and city levels have been reluctant to support homeowner attempts to form HOAs, despite clear legal prescriptions. A woman who lives in Linh Dam explained:

According to extant state regulations, each high-rise building area must have a governance board which represents its residents. […] While we’ve long requested the People’s Committee of the City to intervene, it never notified the developer to form such boards. Of course, this serves developers well as a [governance] boards might affect their benefits or else publicize their management units’ financial reports [exposing mismanagement practices] (interview, 29/7/2013).

Until 2014, legislation required that a representative of the developer attend the constituent assembly of HOAs for them to be officially accredited by district-level governments. In the three NUAs studied, a common developer thwarting strategy was not showing up at these assemblies. Where residents sought to get their HOAs officially accredited by local governments anyway, developers declared them unlawful. In Trung Hoa-Nhan Chinh, residents struggled for eight years to get their first HOA officially recognized, a process which they described as “a long and difficult battle” (interview, 27/6/2013). At the time of our interviews, Linh Dam residents were still fighting to form officially accredited HOAs (interviews, 14/3/2012, 19/6/2013, 20/7/2013).

The 2014 revision of the Law on Housing and subsequent legislative changes (e.g., Decision 02/2016/TT-BXD) have since reduced *de jure* the autonomy of HOAs while strengthening developers’ grip on projects. Policy changes ensconced NUA developers as permanent actors in the administration and management of residential high-rises. They did so by giving them a voting seat on HOA boards and by stipulating that their management units remain in charge of any NUA building with an elevator, which is almost always the case. Moreover, the revised legislation puts the management of building maintenance funds, previously the sole prerogative of HOAs, under the joint responsibility of HOAs and developers’ management units.

## Section 3: Corporate residential administration versus housing activism

While developers might seem to increasingly dominate NUA governance, lived reality in these new residential environments is more complicated. Over the last two decades, the middle classes in Vietnam have, on the one hand, embraced the NUA model of urban development and the benefits it brings them in terms of property investment, lifestyle, social status, and access to modern urban environments and infrastructure (see Duy Luan, 2014; Harms, 2016; Tran, 2018). On the other hand, the market liberalization policies and state business interests underpinning the management of NUAs simultaneously pose a threat to these people’s new material interests, triggering forms of self-protective activism among homeowner groups.

### The triggers for activism: Land use, property rights, and monthly fee disputes

Homeowner-developer opposition began early in the lifecycles of Linh Dam, Trung Hoa-Nhan Chinh, and Van Quan. Over the years, these tensions have tended to center on land use issues, property right ambiguities, and monthly charge increases decreed by property management units.

Land use conflicts arose in the three NUAs studied when new homeowners began to notice important discrepancies between the approved master plans for their developments and the actual built forms and land uses developers implemented. To cite a few examples, Trung Hoa-Nhan Chinh’s residents complained that the developer of their NUA sold sites earmarked for the construction of open public spaces to private entities who used them for commercial buildings and functions. Similarly, a man who has living in Linh Dam lamented the construction of a parking lot in a part of his NUA which used to be a green space: “[The developer] took 600-700 square meters away for a parking. This should have never happened; it’s both unreasonable and ugly” (interview, 8/3/2012). In Van Quan, residents complained about the addition of a 25-story building not in their NUA’s original plans but which, by adding a hundred housing units to the area, heightened density, traffic congestion, and flooding.

A senior municipal planner interviewed for this study indicated that domestic property developers in Vietnam regularly ‘adjust’ land uses during the implementation of projects to increase their profitability (interview, 13/06/2016).^7^ He added that while developers occasionally perform such changes illegally, they are more typically endorsed by state planning authorities. This is, of course, one of the problematic outcomes of the interpenetration of state and business interests in Vietnam’s property development sector, something NUA residents are well aware of as we will see below.

Another source of tensions between homeowners and developers stems from property rights ambiguities. These are particularly common in residential high-rises built in the early 2000s, including Linh Dam and Trung Hoa-Nhan Chinh. Sales contracts and property titles issued to owners of housing units in these buildings did not clearly establish who owns their common areas, opening the way for disputes. A homeowner group in Linh Dam, for instance, has been in a protracted conflict with the developer about ownership of the ground floors of residential high-rises. As explained by a member of that group:

[The developer] wants to keep that floor so he can rent it for grocery stores, cafes, and so on. He has signed decade-long leases and makes tons of money. But in the sale contracts for our apartments, it says that we residents hold collective ownership [*so huu chung*] over that floor. We bought the whole building so how can the developer possibly say he still owns it? It makes no sense! So now we’re fighting to get it back (interview, 14/3/2012).

By far the most common grievance and source of conflict in NUAs concerns the opaque way in which developers set and manage monthly fees. In the three developments studied, over the years, homeowners have seen steep increases in monthly charges for the maintenance of high-rise buildings, urban public spaces, and amenities. For instance, between 2009 and 2012, successive raises ended up doubling monthly charges in Trung Hoa-Nhan Chinh’s high-rise towers. During the late 2000s and early 2010s, such patterns repeated throughout Hanoi’s NUAs, including in Linh Dam and Van Quan.

Faced with these steep raises, homeowners started to question how developers manage revenues and to demand that their management units open up their accounts for inspection. Several interviewees specified that their core concern was not the additional expenditure fee increases represented for their households, but the broader implications of these hikes. “The amount is not an issue,” a woman who owns an apartment in Linh Dam remarked. “What most of us want is public [*cong khai*] and transparent [*minh bach*] management. We want to know what we own, what the developer owns, what revenues our buildings generate, and how this money is used.” (interview, 20/7/2013b). Homeowners in Van Quan and Trung Hoa-Nhan Chinh similarly complained about the opaque practices of management boards in their NUAs and about the fact that they often take decisions directly affecting them and their properties without any consultation or warning.

### Institutional straddling as collective self-protection

These conflicts triggered self-protective responses among the owners of apartments in Linh Dam, Trung Hoa-Nhan Chinh, and Van Quan. In these three places, very few households confronted developers on their own. Instead, aggrieved homeowners have tended to act collectively.

Early on, these groups of housing activists found that local governments at the ward- or district-level were reluctant to intervene in their conflicts with developers. Some interviewees openly vented their frustrations with government officials who they believe to be in political and economic lockstep with actors from the property development sector. A resident of Van Quan thus complained that ward and district governments stay out the conflicts between NUA developers and homeowners “because they received money from the [property] investors” (interview, 7/06/2017). Asked to explain why ward and district governments give developers and property managers free rein in his NUA, a resident of Trung Hoa-Nhan Chinh similarly lamented, “Up there, it’s just a bunch of them shaking hands together” (interview, 22/3/2012).

In this context, NUA housing activists took things into their own hands. One of their first moves was to try to establish one or more HOAs. The hope was that the establishment of self-administration bodies controlled by residents would redress the power imbalance between them and developers. As mentioned above, this was not an easy task. However, when they succeeded, as was the case in Trung Hoa-Nhan Chinh, the new HOAs did provide some ammunition to keep developers in check.

For instance, until the 2014 revision of the Law on Housing, HOAs in Trung Hoa-Nhan Chinh and Van Quan countered monthly fee raises by threatening to dismiss developers from their urban management and building maintenance functions and to replace them with competitors. The HOAs of both developments also addressed petitions to various governmental agencies and called on domestic media to report their stories in order to pressure developers and, ultimately, to settle conflicts (interviews, 14/6/2017 and 22/3/2012).

Preceding the actual collective actions taken by HOAs was a less visible but crucial dimension of residents’ self-protective response: fusing the infrastructure, roles, and leadership of old socialist and new self-managed neighborhood administration bodies. In Linh Dam, Trung Hoa-Nhan Chinh, and Van Quan, a mix of heads and deputy-heads of residents’ groups, local party cell secretaries, and leaders of mass organizations drove homeowner efforts to set up HOAs. In the two developments with officially recognized HOAs at the time of our interviews (i.e., Trung Hoa-Nhan Chinh and Van Quan), these organizations were set up to match the territorial-administrative structure of the existing socialist neighborhood administration apparatus. For instance, Trung Hoa-Nhan Chinh’s largest HOA, which represents 1200 households in eight high-rises, follows the same geographic boundaries as a pre-existing communist party cell. Early on in the lifecycle of this NUA, the territory had been subdivided into eight residents’ groups, each corresponding to a residential high-rise. According to bylaws drafted by homeowners, eight of the 15 seats on the board of the new HOA for this zone are allocated to the heads of the eight residents’ group. This effectively fused not only the administrative territory but also the leadership of the pre-existing neighborhood management organizations set up by the state with that of the new, property self-management body (interviews, 22/3/2012, 29/6/2013, and 29/6/2013). We observed similar patterns in the two other NUAs studied, although homeowners in these developments have established or are starting smaller HOAs comprising two or three buildings (interviews, 18/6/2017 and 20/6/2013).

Discussing the double leadership roles played by heads of residents’ groups, a housing activist told us that integrating the leaders of local state-linked bodies with the board of Trung Hoa-Nhan Chinh’s HOA had been a natural move (interview 29/6/2013). Fusing these roles, he remarked, made the work of the HOAs more convenient [*tien loi*], especially in terms of coordinating actions with the socialist neighborhood administration apparatus. Moreover, this same informant noted, the leaders of state-managed local bodies are generally seen as reputable persons [*nguoi co uy tin*] who serve their communities “with devotion [*tam huyet*] and enthusiasm [*nhiet tinh*]” (interview, 29/6/2013). That is why early on in the process of establishing HOAs, local community members saw them as ideal candidates for the new bodies’ boards. The head of a residents’ group in Van Quan similarly noted that homeowners in her development turned to the leaders of residents’ groups, party cells, and mass organizations for their NUAs boards as they sought to “elect people they can count on” and individuals “proactive in local socio-political organizations” (interview, 9/6/2017).

In heading the new HOAs, leaders of socialist neighborhood administrative bodies may have helped establish the credibility of this new local institution among residents. Just as importantly, they put resources and organizational capacities of their state-managed bodies in the service of local homeowners’ place-based and property interests. In the three NUAs studied, heads of resident groups reached out to their peers working in other developments to learn from their experiences dealing with developer conflict. Acting as the political voice of NUA residents, they called on reluctant local governments (at the ward, district, and even city level) to intervene when developers attempted to impede local efforts to form HOAs. They also used the communication infrastructure of their local state-mandated organization to organize strategy meetings with homeowners. Loudspeakers, bulletin boards, and electronic mailing lists—officially meant to ensure the socialist state’s control over local populations—thus became communication channels between residents defending private property rights (interviews, 29/6/2013, 29/6/2013, 18/6/2017, 20/6/2013).

In Linh Dam, where owners were still struggling to establish HOAs at the time of our interviews, resident group heads have leveraged the support of party cell secretaries and leaders of mass organizations to provide some HOA functions. Even without an HOA, these people coordinated homeowner battles against developers’ successive monthly fee hikes and acted as residents’ representatives in negotiations with management units. When these negotiations failed, resident group leaders used the bulletin boards of residential high-rises (officially meant to disseminate state-sanctioned information) to urge apartment owners to stop paying their monthly charges until further notice (interviews, 19/7/2013, 20/7/2013).

In all three Hanoi NUAs, party cells, mass organizations, and resident groups thus took on roles that seem very far from their official mandate as the transmission belt of the Vietnamese communist party-state. This situation is partly explained by the fact that, as elsewhere in Vietnam, leaders of organizations comprising the socialist neighborhood administration apparatus are themselves local residents. As a Van Quan resident remarked: “They too live in this building so they are also protecting their own rights” (interview, 7/06/2017). Another factor appears to be at play, and it reflects straddler organizations’ tendency to “meld conceptually distinct functions” (Read, 2009: 13). Both local leaders and residents told us that they consider the defense of the place-based and material interests of NUA homeowners to be a duty of local state-linked organizations. As a homeowner of Linh Dam put it, heads of residents’ groups must “protect the residents’ lives [*bao ve doi song nhan dan*]” (interview, 20/07/2013). And this, for many NUA residents, includes warding off the abusive practices of private developers and their management units.

Homeowners in the developments studied did not participate uniformly in conflicts with developers. In the three NUAs, the most prominent housing activists were retirees with a history of active involvement in collective enterprises. Many of these people had been engaged members or leaders of a local state-linked organization before moving to an NUA (either in their previous neighborhood or at their workplace). Conversely, and mirroring their lower participation in the local socio-political life, some working-age homeowners interviewed for this study dismissed disputes between housing activists and developers in their NUA as squabbles of little consequence. Such lack of interest, however, is not pervasive among younger homeowners, many of whom participated in their buildings’ protests, most notably in Van Quan.

## Conclusion

The role played by residents in the everyday governance of commercially built residential developments has received little attention in Southeast Asia, and in Vietnam in particular. This study sought to partially fill this gap by analyzing the micro-governance dynamic of three master-planned communities built on the edges of Hanoi following privatization of residential space production in Vietnam. Drawing on scholarship on the micro-politics of Chinese commercially built residential developments, we have shown that a complex change process is at play in Hanoi’s NUAs. In the three developments studied, middle-class households have simultaneously embraced private property and the lifestyles brought by this new model of urban development and challenged the power and governance responsibilities it has given private developers.

Echoing the literature on China, we have shown that residents’ collective efforts to protect their new place-based interests and property rights against developers’ practices are having a significant impact on the governance dynamic emerging in Hanoi’s NUAs. The specific manner in which this occurs in Hanoi is made possible and shaped by the hybrid territorial-administrative structure established in NUAs, a structure in which the older socialist neighborhood administration apparatus cohabits with new resident private property management groups (HOAs). In the three developments that we studied, groups of homeowners sought to keep developers and their property management companies in check by inventively merging aspects of leadership and action of these two apparently distinct neighborhood governance systems.

We call this mode of political action institutional straddling*.* Local state-linked organizations inherited from socialism not only provided an associational nexus for developing ‘self-protection’ strategies among homeowners, but their leadership and resources merged with the HOAs set up by residents. In the process, activists in the three developments broadened the purposes of residents’ groups, party cells, and local branches of mass organizations well beyond their official mandates. Without relinquishing their official state-defined roles, socialist governance institutions became champions and advocates of new place-based, private property interests in NUAs. The resulting residential governance is both malleable and adaptive, not only reconfiguring the roles and functions of micro-governance institutions inherited from socialism but also destabilizing the newer corporate logics of neighborhood management.

These findings build on interviews conducted throughout the 2010s. In a city marked by rapid socio-political transformations, the dynamics discussed above might only represent a momentary phase. This snapshot of Hanoi’s micro-politics, however, indicates that the idea of institutional straddling can help overcome the limits of binary conceptualizations prevalent in the literature on the privatization of urban space production in East and Southeast Asia. Western-derived notions of private or corporatized neighborhood governance (e.g., McKenzie, 1994), for instance, are geared toward analyzing relatively clear-cut empirical phenomena rather than situations that cross lines between the state, the private sector, and society; between public and private institutions; and between socialist and capitalist structures. The forms of governance emerging in the three developments studied lie on the boundaries of current understandings, justifying more nuanced conceptualizations. Attending to this ambiguous zone opens up a broader perspective on state-society relations, not only in Vietnam and Hanoi, but also across large swath of urban Southeast Asia where privately developed residential areas play a foundational role.

The idea of institutional straddling not only accounts for the complexity of the changes unfolding in Asian cities, but it also points to the importance of situating governance dynamics in their local historical context. With regard to Vietnam, this study supports the view that the *doi moi* reforms—as profound as they might seem—did not “[sweep] away what was there before but instead relate to the persistence, or reworking, of existing power structures” (Gainsborough, 2010: 2). The changing modes of micro-governance that we witnessed in Hanoi’s NUAs indicate that the introduction of a neoliberal or corporate neighborhood management system has not been accompanied by the displacement of micro-local governance institutions inherited from socialism. Instead, the NUA model of urban development, and the preeminence of private developers and property management companies within it, bifurcated local governance dynamics. This marks an evolution of the way residential spaces are governed in the Vietnamese capital city rather than representing an entirely new form of local governance.

Finally, this study has explored emerging forms of governance in three mid-range NUAs, built about two decades ago by state-owned property developers for Vietnamese middle-class households. As mentioned earlier, interviews on which this study built were conducted in the 2010s, when these developments had been inhabited for ten to fifteen years only. Since then the landscape of privately built, master-planned communities has diversified considerably in Hanoi, (see Labbé and Musil, 2017). In the last decade or so, dozens of projects were completed, ranging from highly exclusive gated communities to residential developments in which affordable housing predominates. At the same time, Hanoi’s property sector experienced the growing presence of foreign investors, developers, and property management companies. How has the micro-politics of older NUAs, such as those that we studied, evolved during that period? And what forms does micro-governance takes within the increasingly heterogeneous NUAs built on the territory of Hanoi? By attending to these questions, future research can shed useful light on the variegated forms of local governance triggered by the privatization of urban and residential space production in Vietnam.

## Notes

1. *Khu do thi moi* in Vietnamese.

2. Linh Dam and Van Quan NUA were developed by the Housing and Urban Development Corporation (HUD), and Trung Hoa-Nhan Chinh was developed by the Vietnam Construction and Import–Export company (Vinaconex). While both HUD and Vinaconex then operated under the Ministry of Construction, their involvement was treated as commercial property development.

3. Officially adopted in 1986, these socio-economic reforms introduced market economy principles into the Vietnamese socialist economy and encouraged foreign investments while preserving the one-party, communist political system.

4. This apparatus is not systematically established in Hanoi’s most expensive and exclusive residential developments (see Tran, 2018).

5. Hanoi’s territorial-administrative system is structured in three nested scales: city, districts, and wards.

6. The Vietnamese Fatherland Front is an arm of the Communist Party. It consists of socio-political mass organizations, such as the Ho Chi Minh Communist Youth Union, Women’s Union, and Veterans Association.

7. Domestic media regularly decry such practices. See for instance Ha Noi Moi (2017) and VN Express (2019).
